# Supplementary material for: Couple-Based Carrier Screening: How Gene and Variant Considerations Impact Outcomes
Source: Genes (Basel). 2025 May 30;16(6):671. doi: 10.3390/genes16060671 (PMC12192196; doi:10.3390/genes16060671)
Supplement: Supplementary file 1 [file genes-16-00671-s001.zip › genes-3570662-supplementary.pdf]

Supplementary Table S1: At-risk couples for AR conditions

Gene	Condition(s)	Included in ACMG gene tiers	At-risk couples with genotype	Genotypes[a]	ClinVar (gold star status)[b]	Predicted phenotype(s) for observed genotypes	Disease classification	Impact classification
ACADVL (NM_000018.3)	Very long-chain acyl-CoA dehydrogenase deficiency	Yes	1	P1: c.848T>C, p.(Val283Ala)	Pathogenic (3)	One compound heterozygous individual in literature with non-ketotic hypoglycaemia and hepatic dysfunction at 22 months of age (PMID: 10077518).	Severe/profound	High
				P2: c.1468G>C, p.(Ala490Pro)	Likely pathogenic (3)			
ACSF3 (NM_174917.4)	Combined malonic and methylmalonic aciduria	No	1	P1: c.1672C>T, p.(Arg558Trp)	Pathogenic/Likely pathogenic (2)	Homozygous individuals described with phenotypes ranging from asymptomatic to encephalopathy and recurrent ketoacidosis (PMID: 30740739, 21841779); two homozygous individuals in the gnomAD database v4.1.0 database.	Severe/profound	Low/medium
				P2: c.1672C>T, p.(Arg558Trp)	Pathogenic/Likely pathogenic (2)			
AGXT (NM_000030.3)	Primary hyperoxaluria type 1	Yes	1	P1: c.32C>G, p.(Pro11Arg)	Pathogenic/Likely pathogenic (2)	No known homozygous individuals in literature; 1 homozygote in gnomAD v4.1.0 database.	Severe/profound	Low/medium
				P2: c.32C>G, p.(Pro11Arg)	Pathogenic/Likely pathogenic (2)			
BTD (NM_001370658.1)	Biotinidase deficiency	Yes	1	P1: c.1308A>C,p.(Gln436His)	Pathogenic/Likely pathogenic (2)	Partial biotinidase deficiency is predicted (PMID: 28498829, 27329734).	Severe/profound	Low/medium
				P2: c.1270G>C,p.(Asp424His)	Pathogenic/Likely pathogenic (2)			
			1	P1: c.1270G>C, p.(Asp424His)	Pathogenic/Likely pathogenic (2)	One compound heterozygous individual in literature with partial biotinidase deficiency (PMID: 25967232); individuals compound heterozygous for the p.(Asp424His) variant and a different BTD variant are also described to have partial biotinidase deficiency (PMID: 9654207).	Severe/profound	Low/medium
				P2: c.1429C>T, p.(Pro477Ser)	Pathogenic/Likely pathogenic (2)			
CFTR (NM_000492.3)	Cystic fibrosis	Yes	1	P1: c.1521_1523del, p.(Phe508del)	Pathogenic (4)	Cystic fibrosis	Severe/profound	High
				P2: c.1521_1523del, p.(Phe508del)	Pathogenic (4)			
			1	P1: c.489+1G>T, p.(?)	Pathogenic (4)	Cystic fibrosis	Severe/profound	High
				P2: c.617T>G, p.(Leu206Trp)	Pathogenic (3)			

CYP21A2 (NM_000500.9)	Congenital adrenal hyperplasia	Yes	3	P1: c.955C>T, p.(Gln319*) and whole gene duplication	p.(Gln319*): Pathogenic (2)	Although classic congenital adrenal hyperplasia (CAH) can be predicted in offspring who are homozygous for p.(Gln319*), approximately 84% of p.(Gln319*) alleles are known to be *in cis* with a whole-gene duplication (PMID: 19773403). This significantly reduces the likelihood that one or both partners are true CYP21A2 carriers, and therefore the risk of classic CAH in their offspring is likely to be low.	Severe/profound	Low/medium
				P2: c.955C>T, p.(Gln319*) and whole gene duplication	p.(Gln319*): Pathogenic (2)			
			1	P1: c.955C>T, p.(Gln319*) and whole gene duplication	p.(Gln319*): Pathogenic (2)	Although classic congenital adrenal hyperplasia (CAH) can be predicted in offspring who are compound heterozygous for p.(Gln319*) and c.293-13C>G, approximately 84% of p.(Gln319*) alleles are known to be *in cis* with a whole-gene duplication (PMID: 19773403). This significantly reduces the likelihood that one of the partners is a true CYP21A2 carrier, and therefore the risk of classic CAH in their offspring is likely to be low.	Severe/profound	Low/medium
				P2: c.293-13C>G, p.(?)	Pathogenic/Likely pathogenic (2)			
CYP27A1 (NM_000784.4)	Cerebrotendinous xanthomatosis	Yes	1	P1: c.1537C>T, p.(Arg513Cys)	Pathogenic/Likely pathogenic (2)	No known homozygous individuals in literature; described in multiple affected individuals in the compound heterozygous state (PMID: 28623566, 34012265, 32714376).	Mild/moderate	Low/medium
				P2: c.1537C>T, p.(Arg513Cys)	Pathogenic/Likely pathogenic (2)			
DHCR7 (NM_001360.2)	Smith-Lemli-Opitz syndrome	Yes	1	P1: c.964-1G>C, p.(?)	Pathogenic/Likely pathogenic (2)	Homozygosity commonly reported in literature as resulting in in-utero or early neonatal death (PMID: 29455191, 23293579, 32055014).	Severe/profound	High
				P2: c.964-1G>C, p.(?)	Pathogenic/Likely pathogenic (2)			
GAA (NM_000152.5)	Pompe disease	Yes	1	P1: c.841C>T, p.(Arg281Trp)	Likely pathogenic (3)	No compound heterozygous individuals described; residual GAA enzyme activity has been described for the p.(Arg375Cys) variant (PMID: 36246652); residual enzyme activity typically associated with late-onset disease (PMID: 37759679).	Severe/profound	Low/medium
				P2: c.1123C>T, p.(Arg375Cys)	Uncertain significance (3)			
GJB2 (NM_004004.6)	Nonsyndromic hearing loss	Yes	10	P1: c.109G>A, p.(Val37Ile)	Pathogenic (3)	75% of homozygous individuals have mild to moderate hearing loss (PMID: 31160754).	Mild/moderate	Low/medium
				P2: c.109G>A, p.(Val37Ile)	Pathogenic (3)			
			2	P1: c.101T>C, p.(Met34Thr)	Pathogenic (3)		Mild/moderate	Low/medium

				P2: c.35del, p.(Gly12Valfs*2)	Pathogenic (3)	87% of individuals compound heterozygous for such variants have mild to moderate hearing loss (PMID: 31160754).		
			1	P1: c.101T>C, p.(Met34Thr)	Pathogenic (3)	87% of individuals compound heterozygous for such variants have mild to moderate hearing loss (PMID: 31160754).	Mild/moderate	Low/medium
				P2: c.167del, p.(Leu56Argfs*26)	Pathogenic (3)			
			1	P1: c.109G>A, p.(Val37Ile)	Pathogenic (3)	74% of individuals compound heterozygous for such variants have mild to moderate hearing loss (PMID: 31160754).	Mild/moderate	Low/medium
				P2: c.235del, p.(Leu79Cysfs*3)	Pathogenic (3)			
MYO15A (NM_016239.4)	Nonsyndromic hearing loss	No	1	P1: c.5447T>A, p.(Leu1816*)	Absent in ClinVar	Profound, congenital, neurosensory, nonsyndromic deafness	Mild/moderate	Low/medium
				P2: c.3524dup, p.(Ser1176Valfs*14)	Pathogenic/Likely pathogenic (2)			
NPHS2 (NM_014625.3)	Nephrotic syndrome type 2	No	1	P1: c.686G>A, p.(Arg229Gln)	Conflicting classifications (1)	A compound heterozygous individual (with one of the alleles as Arg229Gln and the other a premature termination variant) has been described in literature with steroid-responsive adult-onset nephrotic syndrome (PMID: 17699384).	Severe/profound	Low/medium
				P2: c.855_856del, p.(Arg286Thrfs*17)	Pathogenic/Likely pathogenic (2)			
PAH (NM_001354304.2)	Phenylketonuria	Yes	1	P1: c.532G>A, p.(Glu178Lys)	Pathogenic (3)	No compound heterozygous individuals for these variants have been described; p.(Glu178Lys) associated with mild hyperphenylalanemia (PMID: 26542770); p.(Gln172His) associated with mild hyperphenylalanemia and classic phenylketonuria (PMID: 26322415, 28982351).	Severe/profound	Low/medium
				P2: c.516G>T, p.(Gln172His)	Likely pathogenic (3)			
POLG (NM_002693.3)	POLG-related disorders	Yes	1	P1: c.3483-4_3497del, p.(?)	Pathogenic/Likely pathogenic (2)	Variable expressivity, incomplete penetrance, and no known genotype-phenotype correlations	Severe/profound	Low/medium
				P2: c.2243G>C, p.(Trp748Ser)	Pathogenic/Likely pathogenic (2)			
			1	P1: c.2209G>C, p.(Gly737Arg)	Pathogenic/Likely pathogenic (2)		Severe/profound	Low/medium
				P2: c.2890C>T, p.(Arg964Cys)	Conflicting classifications (1)			
SBDS (NM_016038.4)	Shwachman-Diamond syndrome	No	1	P1: c.258+2T>C, p.(?)	Pathogenic/Likely pathogenic (2)	Shwachman-Diamond syndrome	Severe/profound	High
				P2: c.183_184delinsCT, p.(Lys62*)	Pathogenic (2)			
SERPINA1 (NM_000295.5)	Alpha1-antitrypsin deficiency	No	1	P1: c.1096G>A, p.(Glu366Lys)	Pathogenic (2)	Alpha1-antitrypsin deficiency; 2% risk of severe liver disease in childhood (GeneReviews - Alpha-1 Antitrypsin Deficiency).	Severe/profound	Low/medium
				P2: c.1096G>A, p.(Glu366Lys)	Pathogenic (2)			

SMN1 (NM_000344.4)	Spinal muscular atrophy	Yes	1	P1: 1 gene deletion	Not applicable	Spinal muscular atrophy	Severe/profound	High
				P2: 1 gene deletion				
TYR (NM_000372.5)	Oculocutaneous albinism type IA and IB	Yes	1	P1: c.1217C>T, p.(Pro406Leu)	Pathogenic/Likely pathogenic (2)	Homozygous individuals with oculocutaneous type 1B OCA have been described in literature (PMID: 25216246, 1903591, 18463683); also, several homozygous individuals are observed in the gnomAD v4.1.0 database.	Mild/moderate	Low/medium
				P2: c.1217C>T, p.(Pro406Leu)	Pathogenic/Likely pathogenic (2)			
			1	P1: c.1205G>A, p.(Arg402Gln); c.575C>A, p.(Ser192Tyr)	Likely pathogenic (2)	Compound heterozygous individuals with ocular albinism and oculocutaneous albinism described in literature (PMID: 31719542, 37460203).	Mild/moderate	Low/medium
				P2: c.823G>T, p.(Val275Phe)	Pathogenic/Likely pathogenic (2)			
			1	P1: c.1205G>A, p.(Arg402Gln); c.575C>A, p.(Ser192Tyr)	Likely pathogenic (2)	Compound heterozygous individuals with ocular albinism, without skin hypopigmentation, described in literature (PMID: 31719542, 18326704).	Mild/moderate	Low/medium
				P2: c.1118C>A, p.(Thr373Lys)	Pathogenic (2)			
			1	P1: c.1217C>T, p.(Pro406Leu)	Pathogenic/Likely pathogenic (2)	No known compound heterozygous individuals in literature.	Mild/moderate	Low/medium
				P2: c.1037-7T>A, p.(?)	Pathogenic/Likely pathogenic (2)			
			1	P1: c.1217C>T, p.(Pro406Leu)	Pathogenic/Likely pathogenic (2)	No known compound heterozygotes in literature.	Mild/moderate	Low/medium
				P2: c.1205G>A, p.(Arg402Gln); c.575C>A, p.(Ser192Tyr)	Likely pathogenic (2)			
			1	P1: (HOMOZYGOUS) c.1205G>A, p.(Arg402Gln); c.575C>A, p.(Ser192Tyr)	Likely pathogenic (2)	Compound heterozygous individuals with ocular albinism (PMID: 18326704).	Mild/moderate	Low/medium
				P2: c.1037-7T>A, p.(?)	Pathogenic/Likely pathogenic (2)			

[a]All individuals are heterozygous unless otherwise indicated; [b]ClinVar database accessed 21 August 2024; P1: partner 1; P2: partner 2

Supplementary Table S2: At-risk couples for X-linked conditions

Sex	Age	Gene	Condition	Genotype	ClinVar (gold star status)[a]	Predicted phenotype	Disease classification	Impact classification
F	29	*FMR1*	Fragile X syndrome	30,83	Not applicable	>65 repeats (higher risk of expansion into full mutation range)	Severe/profound	High
F	34	*FMR1*	Fragile X syndrome	30,102	Not applicable		Severe/profound	High
F	32	*FMR1*	Fragile X syndrome	30,73	Not applicable		Severe/profound	High
F	25	*FMR1*	Fragile X syndrome	30,68	Not applicable		Severe/profound	High
F	28	*FMR1*	Fragile X syndrome	19,55	Not applicable	<65 repeats (≤5% risk of expansion into full mutation range irrespective of AGG interrupts)	Severe/profound	Low/medium
F	32	*FMR1*	Fragile X syndrome	30,56	Not applicable		Severe/profound	Low/medium
F	33	*FMR1*	Fragile X syndrome	31,55	Not applicable		Severe/profound	Low/medium
F	35	*FMR1*	Fragile X syndrome	29,56	Not applicable		Severe/profound	Low/medium
F	44	*FMR1*	Fragile X syndrome	46,57	Not applicable		Severe/profound	Low/medium
F	35	*FMR1*	Fragile X syndrome	32,59	Not applicable		Severe/profound	Low/medium
F	35	*FMR1*	Fragile X syndrome	31,56	Not applicable		Severe/profound	Low/medium
F	37	*FMR1*	Fragile X syndrome	29,64	Not applicable		Severe/profound	Low/medium
F	30	*FMR1*	Fragile X syndrome	30,56	Not applicable		Severe/profound	Low/medium
F	32	*FMR1*	Fragile X syndrome	33,63	Not applicable		Severe/profound	Low/medium
F	36	*FMR1*	Fragile X syndrome	30,55	Not applicable		Severe/profound	Low/medium
F	32	*DMD*	Duchenne/Becker muscular dystrophy	HET exon 17-20 deletion (NM_004006.3)	Not applicable	In-frame deletion not described in literature; in-frame deletions involving exons 3-20 are commonly associated with Duchenne muscular dystrophy (PMID: 33238405).	Severe/profound	High
F	35	*DMD*	Duchenne/Becker muscular dystrophy	HET exon 3-9 deletion (NM_004006.3)	Not applicable	In-frame deletion associated with Becker muscular dystrophy in literature (PMID: 27009627, 7991131, 8429320, 8317478).	Severe/profound	High
F	38	*DMD*	Duchenne/Becker muscular dystrophy	HET NM_004006.3:c.10224-2A>G, p.(?)	Likely pathogenic (1)	Novel canonical acceptor splice site variant; skipping of the adjacent exon 71 is expected to result an in-frame change.	Severe/profound	High
F	36	*DMD*	Duchenne/Becker muscular dystrophy	HET exon 17-44 duplication (NM_004006.3)	Not applicable	In-frame duplication associated with Becker muscular dystrophy in two individuals in the edystrophin database (edystrophin.genouest.org).	Severe/profound	High
F	34	*GLA*	Fabry disease	HET NM_000169.3:c.640-801G>A, p.(?)	Pathogenic (2)	Reported in literature in association with late-onset, incompletely penetrant hypertrophic cardiomyopathy in male and female individuals (PMID: 20031620, 25762495, 34067605); renal and ocular abnormalities also described (PMID: 20821055).	Mild/moderate	Low/medium
F	28	*GLA*	Fabry disease	HET NM_000169.3:c.870G>C, p.(Met290Ile)	Pathogenic/Likely pathogenic (2)	Reported in literature in female individuals with either classic or late-onset Fabry disease (PMID: 33527381, 28728877, 31519519, 33907643); one	Mild/moderate	Low/medium

						73 year old male individual with low alpha-galactosidase A enzyme activity levels and no known past medical history (PMID: 33907643); 2 hemizygotes in gnomAD v4.1.0 database for a different cDNA change leading to same missense change, c.870G>A, p.(Met290Ile).		
F	34	*GLA*	Fabry disease	HET NM_000169.3:c.1088G>A, p.(Arg363His)	Pathogenic/Likely pathogenic (2)	Reported in literature in male and female individuals with late-onset Fabry disease (PMID: 26937405, 34199132, 33437642, 11668641); also reported in some individuals with classic Fabry disease (PMID: 28360401, 12175777).	Mild/moderate	Low/medium
F	34	*F8*	Haemophilia A	HET exon 1-22 deletion (NM_000132.4)	Not applicable	Severe haemophilia (PMID: 22906111, 18371163, 18665854, 27766059, 29296726)	Severe/profound	High
F	33	*F8*	Haemophilia A	HET exon 1-22 deletion (NM_000132.4)	Not applicable		Severe/profound	High
F	33	*GJB1*	X-linked Charcot-Marie-Tooth disease	HET NM_000166.6:c.-103C>T, p.(?)	Pathogenic/Likely pathogenic (2)	Charcot-Marie-Tooth disease	Mild/moderate	Low/medium

[a]ClinVar database accessed 21 August 2024; HET: heterozygous

Supplementary Table S3: Secondary findings for autosomal dominant phenotypes

	Sex	Age	Gene	Variant	ClinVar classification[a]
1	M	36	*ATM*	HET NM_000051.4:c.170G>A, p.(Trp57*)	Pathogenic
2	F	34	*ATM*	HET NM_000051.4:c.8786+1G>A, p.(?)	Pathogenic
3	M	29	*ATM*	HET NM_000051.4:c.2413C>T, p.(Arg805*)	Pathogenic
4	M	40	*ATM*	HET NM_000051.4:c.5228C>T, p.(Thr1743Ile)	Pathogenic/Likely pathogenic
5	F	30	*ATM*	HET NM_000051.4:c.331+5G>A, p.(?)	Pathogenic/Likely pathogenic
6	F	36	*ATM*	HET NM_000051.4:c.3756T>A, p.(Tyr1252*)	Pathogenic/Likely pathogenic
7	F	30	*ATM*	HET NM_000051.4:c.2250+2T>C, p.(?)	Pathogenic/Likely pathogenic
8	M	32	*ATM*	HET NM_000051.4:c.5449_5450dup, p.(Gly1818Glufs*11)	Absent from ClinVar
9	F	28	*LDLR*	HET NM_000527.5:c.682G>T, p.(Glu228*)	Pathogenic
10	M	42	*LDLR*	HET NM_000527.5:c.260G>A, p.(Trp87*)	Pathogenic
11	M	43	*LDLR*	HET NM_000527.5:c.260G>A, p.(Trp87*)	Pathogenic
12	F	38	*LDLR*	HET NM_000527.5:c.1444G>A, p.(Asp482Asn)	Pathogenic/Likely pathogenic
13	M	49	*LDLR*	HET NM_000527.5:c.1691A>G, p.(Asn564Ser)	Pathogenic/Likely pathogenic
14	M	31	*LDLR*	HET NM_000527.5:c.502G>A, p.(Asp168Asn)	Pathogenic/Likely pathogenic
15	F	36	*LDLR*	HET NM_000527.5:c.761A>C, p.(Gln254Pro)	Pathogenic/Likely pathogenic
16	F	34	*LDLR*	HET NM_000527.5:c.782G>T, p.(Cys261Phe)	Pathogenic/Likely pathogenic
17	F	42	*LDLR*	HET NM_000527.5:c.1195G>A, p.(Ala399Thr)	Conflicting classifications
18	M	40	*LDLR*	HET NM_000527.5:c.1747C>T, p.(His583Tyr)	Conflicting classifications
19	M	32	*LDLR*	HET NM_000527.5:c.1747C>T, p.(His583Tyr)	Conflicting classifications
20	F	37	*LDLR*	HET NM_000527.5:c.1241T>G, p.(Leu414Arg)	Uncertain significance
21	M	29	*TTN*	HET NM_001267550.2:c.106945G>T, p.(Glu35649*)	Likely pathogenic
22	F	37	*TTN*	HET NM_001267550.2:c.83126G>A, p.(Trp27709*)	Likely pathogenic
23	M	37	*TTN*	HET NM_001267550.2:c.56154G>A, p.(Trp18718*)	Likely pathogenic
24	F	36	*TTN*	HET NM_001267550.2:c.52825C>T, (p.Gln17609*)	Likely pathogenic
25	F	33	*TTN*	HET NM_001267550.2:c.89861G>A, p.(Trp29954*)	Likely pathogenic
26	F	47	*TTN*	HET NM_001267550.2:c.54636T>G, p.(Tyr18212*)	Likely pathogenic
27	M	30	*TTN*	HET NM_001267550.2:c.67495C>T, p.(Arg22499*)	Pathogenic/Likely pathogenic
28	M	31	*TTN*	HET NM_001267550.2:c.59926+1G>A, p.(?)	Pathogenic/Likely pathogenic
29	M	37	*TTN*	HET NM_001267550.2:c.76115dup, p.(Asn25372Lysfs*5)	Conflicting classifications
30	F	39	*TTN*	HET NM_001267550.2:c.40723+1G>T, p.(?)	Conflicting classifications
31	M	34	*TTN*	HET NM_001267550.2:c.76639del, p.(Val25547Trpfs*10)	Absent from ClinVar
32	F	35	*TTN*	HET NM_001267550.2:c.102331del, p.(Ile34111Serfs*13)	Absent from ClinVar
33	M	36	*TTN*	HET NM_001267550.2:c.12557del, p.(Pro4186Glnfs*6)	Absent from ClinVar
34	F	34	*TTN*	HET NM_001267550.2:c.105474_105477del, p.(Thr35159*)	Absent from ClinVar
35	F	28	*TTN*	HET NM_001267550.2:c.90130_90131del, p.(Lys30044Aspfs*11)	Absent from ClinVar
36	F	30	*TTN*	HET NM_001267550.2:c.67458T>A, p.(Tyr22486*)	Absent from ClinVar
37	F	34	*TTN*	HET NM_001267550.2:c.73545_73546del, p.(Arg24515Serfs*18)	Absent from ClinVar
38	F	34	*TTN*	HET NM_001267550.2:c.83743C>T, p.(Gln27915*)	Uncertain significance

[a]ClinVar database accessed 21 August 2024; HET: heterozygous

Supplementary Table S4: Secondary findings for autosomal recessive phenotypes

	Sex	Age	Gene	Variant 1	Variant 2	Predicted phenotype(s)
1	F	40	*GJB2*	HET NM_004004.6:c.101T>C, p.(Met34Thr)	HET NM_004004.6:c.35del, p.(Gly12Valfs*2)	Hearing loss is likely to be bilateral, mild to moderate, childhood onset (<18 years of age), and slowly progressive over time (PMID: 31160754).
2	F	41	*GJB2*	HOM NM_004004.6:c.109G>A, p.(Val37Ile)		
3	F	34	*GJB2*	HOM NM_004004.6:c.109G>A, p.(Val37Ile)		
4	M	35	*GJB2*	HOM NM_004004.6:c.109G>A, p.(Val37Ile)		
5	M	35	*SERPINA1*	HET NM_000295.5:c.1096G>A, p.(Glu366Lys)	HET NM_000295.5:c.863A>T, p.(Glu288Val)	If these variants are on different alleles (in trans), this increases the risk of chronic obstructive pulmonary disease (COPD); tobacco smoking is a known risk factor for development of COPD.
6	M	36	*SERPINA1*	HET NM_000295.5:c.1096G>A, p.(Glu366Lys)	HET NM_000295.5:c.863A>T, p.(Glu288Val)	
7	F	34	*SERPINA1*	HET NM_000295.5:c.1096G>A, p.(Glu366Lys)	HET NM_000295.5:c.863A>T, p.(Glu288Val)	
8	M	32	*SLC12A3*	HET NM_001126108.2:c.2864G>A, p.(Arg955Gln)	HET NM_001126108.2:c.1928C>T, p.(Pro643Leu)	If these variants are on different alleles (in trans), this could result in Gitelman syndrome (GS). GS is a renal tubular salt-wasting disorder characterised by hypokalemic metabolic alkalosis with hypomagnesemia and hypocalciuria. Most patients have onset of symptoms as adults, but some present in childhood. Clinical features can include transient periods of muscle weakness and tetany, abdominal pains, and chondrocalcinosis.
9	M	34	*SLC12A3*	HET NM_001126108.2:c.2864G>A, p.(Arg955Gln)	HET NM_001126108.2:c.1928C>T, p.(Pro643Leu)	
10	F	33	*ETFDH*	HOM NM_004453.4:c.152G>A, p.(Arg51Gln)		This could result in late-onset multiple acyl-coa dehydrogenase deficiency (MADD). Clinical features of the late-onset form of this condition can include proximal muscle weakness, exercise intolerance, raised creatine kinase levels, and/or muscle pain. Signs and symptoms may become apparent at any time from infancy to adulthood. This specific variant has been observed as compound heterozygous genotypes in multiple individuals with late-onset MADD (PMID: 24357026, 29336361, 34819910, 35342266); however, homozygotes have not been described.

11	F	36	*NPC1*	HET NM_000271.5:c.2621A>T, p.(Asp874Val)	HET NM_000271.5:c.3019C>G, p.(Pro1007Ala)	If these variants are on different alleles (in trans), this could result in Niemann-Pick disease type C (NPC), an autosomal recessive lysosomal storage disorder. Age of clinical presentation can vary from early infancy to adulthood. Adult-onset NPC is dominated by neurologic manifestations, including vertical supranuclear gaze palsy, ataxia, dystonia, cognitive decline, psychiatric disorders, and epilepsy. The combination of these two NPC1 variants has not been described in the literature. However, both p.(Pro1007Ala) and p.(Asp874Val) variants, in conjunction with other disease-causing variants, have been described in individuals with adult-onset disease (PMID: 26666848).
12	M	36	*POLG*	HET NM_002693.3:c.2209G>C, p.(Gly737Arg)	HET NM_002693.3:c.2740A>C, p.(Thr914Pro)	[Proven to be in trans through parental testing] This could result in a POLG-related condition, which comprise a continuum of overlapping phenotypes with ages of onset ranging from infancy to late adulthood. Clinical features in adult-onset disease may include progressive external ophthalmoplegia, myopathy, peripheral neuropathy, ataxia, and cognitive impairment.
13	M	38	*AGL*	HET NM_000642.3:c.4459C>T, p.(Arg1487*)	HET NM_000642.3:c.664+3A>G, p.(?)	If these variants are on different alleles (in trans), this could result in glycogen storage disease type III (GSD III). GSD III is an inborn error of metabolism of variable severity that primarily affects the liver, heart, and skeletal muscle. Clinical features in adults can include hepatic cirrhosis, cardiac hypertrophy and/or cardiomyopathy, and skeletal muscle weakness and wasting.
14	F	43	*ATP7B*	HET NM_000053.4:c.2122-2A>G, p.(?)	HET NM_000053.4:c.3646G>A, p.(Val1216Met)	If these variants are on different alleles (in trans), this could result in Wilson's disease, a disorder of copper metabolism. Clinical features in untreated disease can include hepatic, neurologic, and psychiatric disturbances. Age of onset and range and severity of manifestations are variable. Untreated Wilson's disease has been associated with subfertility and

						spontaneous miscarriage (PMID: 31179293, 33549482).
15	F	33	*VWF*	HOM NM_000552.5:c.2561G>A, p.(Arg854Gln)		This genotype is associated with type 2N Von Willebrand disease (VWD) (PMID: 22875612, 15670054). Clinical features of type 2N VWD mimic those seen in mild hemophilia A and include excessive bleeding at the time of surgery or procedures.

HET: heterozygous; HOM: homozygous

Supplementary Table S5: Carrier screen gene list

AAAS	*ATP7B*	*COL7A1*	*ERCC3*	*GHRHR*	*LAMA2*	*NAGS*	*PMM2*	*SLC19A2*	*TTN*
ABCA12	*ATP8B1*	*CPS1*	*ERCC4*	*GJB1**	*LAMA3*	*NBN*	*PNPO*	*SLC22A5*	*TTPA*
ABCB11	*ATRX**	*CPT1A*	*ERCC5*	*GJB2*	*LAMB3*	*NDRG1*	*POLG*	*SLC25A13*	*TYMP*
ABCB4	*BBS1*	*CPT2*	*ERCC6*	*GJB6*	*LAMC2*	*NDUFAF5*	*POLH*	*SLC25A15*	*TYR*
ABCC6	*BBS10*	*CRB1*	*ERCC8*	*GLA**	*LCA5*	*NDUFS4*	*POMGNT1*	*SLC25A20*	*TYRP1*
ABCC8	*BBS12*	*CTNS*	*ESCO2*	*GLB1*	*LDLR*	*NDUFS6*	*POR*	*SLC26A2*	*UGT1A1*
*ABCD1**	*BBS2*	*CTSC*	*ETFA*	*GLDC*	*LDLRAP1*	*NEB*	*PPT1*	*SLC26A3*	*USH1C*
ACAD9	*BBS4*	*CTSD*	*ETFB*	*GLE1*	*LHCGR*	*NEU1*	*PREPL*	*SLC26A4*	*USH2A*
ACADM	*BBS9*	*CTSK*	*ETFDH*	*GNE*	*LIFR*	*NPC1*	*PROP1*	*SLC35A3*	*VPS13A*
ACADVL	*BCKDHA*	*CYBA*	*ETHE1*	*GNPTAB*	*LIPA*	*NPC2*	*PRPS1**	*SLC37A4*	*VPS13B*
ACAT1	*BCKDHB*	*CYBB**	*EVC*	*GNPTG*	*LOXHD1*	*NPHP1*	*PSAP*	*SLC39A4*	*VPS45*
ACOX1	*BCS1L*	*CYP11B1*	*EVC2*	*GNS*	*LPL*	*NPHS1*	*PTS*	*SLC45A2*	*VPS53*
ACSF3	*BLM*	*CYP11B2*	*EXOSC3*	*GORAB*	*LRPPRC*	*NPHS2*	*PUS1*	*SLC6A8**	*VRK1*
ADA	*BSND*	*CYP17A1*	*EYS*	*GUCY2D*	*LYST*	*NR0B1**	*RAB23*	*SLC7A7*	*VSX2*
ADAMTS2	*BTD*	*CYP19A1*	*F8**	*GUSB*	*MAN2B1*	*NR2E3*	*RAG1*	*SMARCAL1*	*VWF*
ADGRG1	*BTK**	*CYP1B1*	*F9**	*HADHA*	*MCOLN1*	*NTRK1*	*RAG2*	*SMN1*	*WAS**
AGA	*CANT1*	*CYP21A2*	*FAH*	*HADHB*	*MECP2**	*OAT*	*RAPSN*	*SMPD1*	*WNT10A*
AGL	*CAPN3*	*CYP27A1*	*FAM161A*	*HAX1*	*MED17*	*OCRL**	*RARS2*	*SRD5A2*	*WRN*
AGPS	*CASQ2*	*CYP27B1*	*FANCA*	*HBA1*	*MESP2*	*OPA3*	*RDH12*	*ST3GAL5*	*XPA*
AGXT	*CBS*	*DBT*	*FANCC*	*HBA2*	*MFSD8*	*OTC**	*RLBP1*	*STAR*	*XPC*
AIRE	*CC2D1A*	*DCLRE1C*	*FANCG*	*HBB*	*MKKS*	*PAH*	*RMRP*	*SUCLA2*	*ZFYVE26*
ALDH3A2	*CCN6*	*DDB2*	*FH*	*HEXA*	*MKS1*	*PANK2*	*RNASEH2C*	*SUMF1*	
ALDH7A1	*CDH23*	*DHCR7*	*FKRP*	*HEXB*	*MLC1*	*PC*	*RPE65*	*SURF1*	
ALDOB	*CEP290*	*DHDDS*	*FKTN*	*HGSNAT*	*MLYCD*	*PCCA*	*RPGRIP1L*	*TAT*	
ALG6	*CERKL*	*DKC1**	*FMR1**	*HJV*	*MMAA*	*PCCB*	*RS1**	*TCIRG1*	
ALMS1	*CFTR*	*DLD*	*G6PC*	*HLCS*	*MMAB*	*PCDH15*	*RTEL1*	*TECPR2*	
ALPL	*CHM**	*DMD**	*GAA*	*HMGCL*	*MMACHC*	*PDHA1**	*SACS*	*TFR2*	
AMT	*CHRNE*	*DNAH5*	*GALC*	*HMOX1*	*MMADHC*	*PDHB*	*SAMD9*	*TGM1*	
AP1S1	*CHRNG*	*DNAI1*	*GALE*	*HPD*	*MMUT*	*PEPD*	*SAMHD1*	*TH*	
AQP2	*CIITA*	*DNAI2*	*GALK1*	*HPS1*	*MOCS1*	*PET100*	*SBDS*	*TMC1*	
*AR**	*CLN3*	*DNAL1*	*GALNS*	*HPS3*	*MPI*	*PEX1*	*SEPSECS*	*TMEM216*	
ARG1	*CLN5*	*DOK7*	*GALNT3*	*HPS4*	*MPL*	*PEX10*	*SERPINA1*	*TPO*	
ARSA	*CLN6*	*DPYD*	*GALT*	*HSD17B3*	*MPV17*	*PEX12*	*SGCA*	*TPP1*	
ARSB	*CLN8*	*DYSF*	*GAMT*	*HSD17B4*	*MRE11*	*PEX2*	*SGCB*	*TREX1*	
ASL	*CLRN1*	*EDA**	*GBA1*	*HSD3B2*	*MTHFR*	*PEX6*	*SGCD*	*TRIM32*	
ASNS	*CNGA3*	*EDAR*	*GBE1*	*HYLS1*	*MTM1**	*PEX7*	*SGCG*	*TRIM37*	
ASPA	*CNGB3*	*EIF2AK3*	*GCDH*	*IDS**	*MTRR*	*PFKM*	*SGSH*	*TRMU*	
ASS1	*COL11A2*	*EIF2B5*	*GCH1*	*IDUA*	*MTTP*	*PHGDH*	*SKIC3*	*TSEN54*	
ATM	*COL4A3*	*ELP1*	*GDF5*	*IL2RG**	*MYO15A*	*PIGN*	*SLC12A3*	*TSFM*	
ATP6V1B1	*COL4A4*	*EMD**	*GFM1*	*IVD*	*MYO7A*	*PKHD1*	*SLC12A6*	*TSHB*	
*ATP7A**	*COL4A5**	*ERCC2*	*GH1*	*KCNJ11*	*NAGLU*	*PLA2G6*	*SLC17A5*	*TSHR*	

* X-linked gene
